# Investigation of the effects of recombinant and urinary FSH used for ovulation induction on pregnancy results in an experimental animal model in terms of endometrial receptivity markers

**DOI:** 10.1186/s12884-026-08706-0

**Published:** 2026-02-03

**Authors:** Hayrunnisa Yeşil Sarsmaz, Yıldız Acar, Seren Gülşen Gürgen, Kemal Sarsmaz

**Affiliations:** 1 Department of Histology and Embryology, Faculty of Health Sciences, Manisa Celal Bayar University, Manisa, Turkey; 2 Department of Midwifery, Faculty of Health Sciences, Manisa Celal Bayar University, Manisa, Turkey; 3 Department of Histology and Embryology, Vocational School of Health Services, Manisa Celal Bayar University, Manisa, Turkey; 4 Department of Obstetrics and Gynecology, Faculty of Medicine, Manisa Celal Bayar University, Manisa, Turkey

**Keywords:** Endometrial receptivity, Laminin, Integrin alpha v beta 3, LIF, Recombinant FSH, Urinary FSH

## Abstract

**Background:**

The aim of this study was to compare recombinant and urinary follicle stimulating hormone (FSH) with each other in terms of endometrial receptivity for the purpose of controlled ovarian stimulation.

**Methods:**

Twenty-four female albino mice (Mus musculus, C/C; 6–8 weeks, 18–22 g) and six males (8–10 weeks) were divided into four groups: control (no mating), spontaneous mating, urinary FSH (uFSH), and recombinant FSH (rFSH). Females in estrus received 5 IU of uFSH or rFSH, followed by mating after 48 h. Implantation sites were evaluated using histopathology and immunohistochemistry with anti-LIF, anti-Laminin, and integrin αVβ3 antibodies. LIF levels in serum collected from the tail vein were measured using ELISA.

**Results:**

The Tukey multiple comparison test showed significant group differences in Laminin and Integrin αVβ3 staining intensity (*p* < 0.001). rFSH treatment significantly increased Laminin and Integrin αVβ3 expression compared with both uFSH and spontaneous conception groups (*p* < 0.001), while uFSH also showed higher levels than the spontaneous group (*p* < 0.001). For LIF, no difference was found between the control and spontaneous groups (*p* > 0.05), but both rFSH and uFSH groups exhibited higher expression than the spontaneous group, with the highest levels in the rFSH group (*p* < 0.001).

**Conclusion:**

rFSH treatment was associated with the greatest enhancement of endometrial receptivity markers, both immunohistochemically and biochemically, suggesting that rFSH may exert a more favorable effect on implantation potential compared with uFSH.

**Graphical Abstract:**

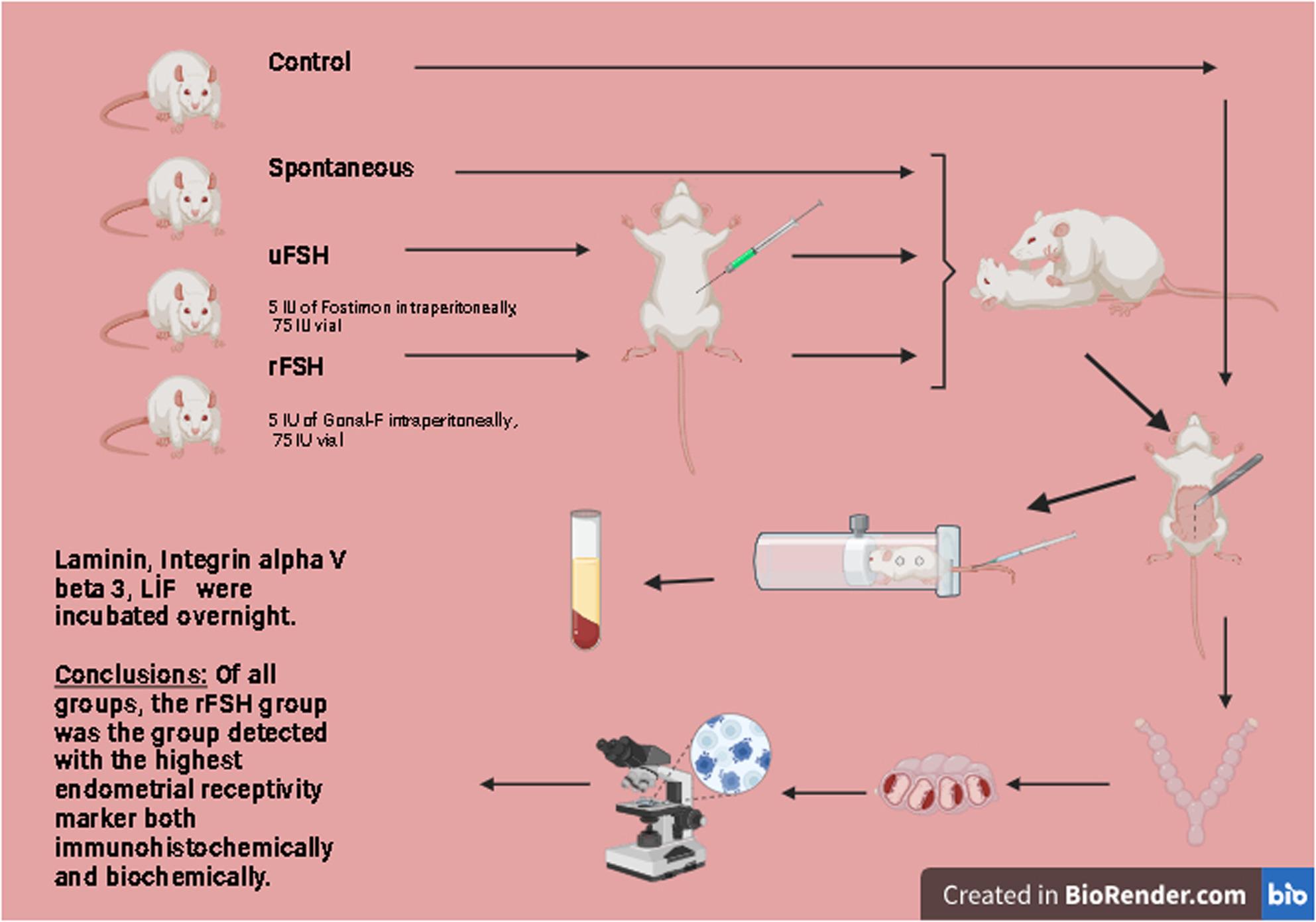

**Supplementary Information:**

The online version contains supplementary material available at 10.1186/s12884-026-08706-0.

## Introduction

Today, infertility is one of the biggest clinical problems, and 10–15% of newly married couples are diagnosed with infertility within a short time [1]. The commonest causes of infertility in women are tubal-peritoneal, ovulatory and unknown causes [2]. FSH is a gonadotrophin which is secreted along with LH from the pituitary gland and regulates ovary function. It is a hormone which is necessary for normal growth, sexual development and reproduction, and is under the control of gonadotropin-releasing hormone (GnRH), which is produced in the hypothalamus and released in response to circulating estrogen and progesterone levels. FSH interacts with the receptors on the ovarian follicles, and is a major survival factor for maturing follicles [3]. LH and FSH are secreted by gonadotropic cells in the pituitary gland as a response to pulsatile hypothalamic GnRH secretion, and control the production of hormones and gametes in the reproductive organs [4].

Stimulation of the ovaries with clomiphene citrate and gonadotropins is called ovulation induction or controlled ovarian stimulation [5]. Controlled ovarian hyperstimulation is an inseparable part of in vitro fertilization and embryo transfer treatment. With the application of exogenic gonadotropins, follicular strengthening and oocytes with increased productivity are obtained. With this aim, different FSH preparations were administered in various ovarian stimulation regimens with variable clinical results [6].

Until recently, the main exogenic source of FSH for therapeutic use was the urine of postmenopausal women. With the advent of recombinant DNA technology, two pure FSH preparations have become available, and it is thought that the purity and in vivo bioactivity of recombinant FSH, follitropin-alpha and follitropin-beta, which do not have LH activity or foreign human proteins, provide safety, efficacy, and tolerability advantages over urine-derived FSH [7, 8].Nevertheless, urinary FSH may be preferred, since studies in IVF cycles have demonstrated higher numbers of fertilized oocytes, higher fertilization rates, better-quality embryos, and increased numbers of frozen embryos when uFSH is used. There are studies on IVF cycles in which a higher fertilization rate, embryos of higher quality and a greater number of frozen embryos have been obtained [9, 10]. A topic on which researchers are in agreement, however, is that the cost of ovulation induction with rFSH is greater than with uFSH [11].

Endometrial receptivity is a mutual interaction between blastocyst and uterus in the period of the implantation window. This receptivity includes the loss of inhibitory components that can act as a barrier for an adherent embryo and the acquisition of adhesion ligands. Embryo implantation in many ways represents the most critical step in the reproductive process. Successful implantation necessitates a receptive endometrium and a normal and functional embryo at the blastocyst stage of development, and also a synchronized dialog between maternal and embryonic tissues. The implantation process can be divided into three stages: apposition, attachment and invagination. Implantation failure continues to be a medically unsolved problem and is accepted as the main reason for infertility in healthy women. Current evidence indicates that embryo quality is responsible for the majority of implantation failures [12]. Inadequate uterine receptivity is estimated to account for approximately two-thirds of implantation failures, whereas embryonic factors are responsible for about one-thirds [13, 14].

Leukemia Inhibitory Factor (LIF) is an interleukin 6 class cytokine which affects cell growth by inhibiting differentiation. When LIF levels fall, cells differentiate [15]. LIF is expressed in the trophectoderm of the developing embryo, and at the same time, and its receptor, LIFR, is also expressed throughout the inner cell mass. Before implantation, LIF is present in the uterine glands and the luminal epithelium. Its role at this stage involves preparing the uterine lining for the arrival of the blastocyst by modulating endometrial receptivity [16–18].

Laminin expression is tightly regulated and varies spatially and temporally during implantation. The dynamic expression of laminins in the uterus coincides with the “window of implantation,” the period during which the endometrium becomes receptive to the blastocyst. In the pre-implantation stage, laminin expression is primarily observed in the uterine epithelium and basement membrane. Laminins at this stage serve to maintain epithelial integrity and prepare the uterine lining for the impending attachment of the blastocyst [19, 20].

The expression of integrin αvβ3 in the uterus is closely linked to the window of implantation, a period of heightened endometrial receptivity that occurs in the mid-secretory phase of the menstrual cycle. Several studies have demonstrated that integrin αvβ3 is expressed predominantly in the luminal and glandular epithelium of the endometrium during this window [21, 22]. The presence of integrin αvβ3 at this stage is thought to prepare the endometrium for the attachment of the blastocyst [23].

The aim of this study was to examine the results concerning endometrial receptivity of recombinant and urinary FSH using controlled ovarian stimulation. In pregnancies obtained as a result of controlled ovarian stimulation with uFSH and rFSH injected intraperitoneally into mice, anti-LIF, anti-Laminin and anti-Integrin alpha V beta 3 primary immunoreactivity was examined in terms of endometrial receptivity, compared to pregnancies obtained as a result of spontaneous mating. The data were examined histopathologically, immunohistochemically and biochemically.

## Material and method

### Study design and animals

Manisa Celal Bayar University Faculty of Medicine Animal Experiments Ethical Committee approval was obtained for this randomized, controlled experimental study (approval no: 77.637.435; 22.12.2020). All protocols were reviewed and approved by the institutional ethics committee for animal experimentation. Animal experiments complied with the ARRIVE guidelines and were conducted in accordance with the National Institutes of Health Guide for the Care and Use of Laboratory Animals (NIH Publications No. 8023, revised 1978).

Animals were randomly assigned to groups using simple randomization, which involved assigning a random number to each subject, ranking them, and distributing them accordingly. An induced experimental animal model was established using 24 albino female mice (Mus musculus, BALB/c strain; 18–22 g, 6–8 weeks old, nulliparous, and not previously used in any experiments) and six male mice (8–10 weeks old; one male per three randomly selected females for mating). All mice were clinically healthy and maintained under specific pathogen-free (SPF) conditions. The animals were wild-type (non-genetically modified) and had not been subjected to any experimental procedures prior to inclusion in the study. All animals were obtained from the Laboratory Animals Department, Faculty of Medicine, Manisa Celal Bayar University.

### Animal uterus tissue sample collection

After three weeks of observation, when it was confirmed that the mice were in regular menstrual cycle, they were randomly divided into four groups.

- Group 1: the control group, not allowed to mate (n: 6 females).
- Group 2: group allowed spontaneous mating (n: 6 females; 2 males were added for mating).
- Group 3: experimental group, given 5 IU of Fostimon intraperitoneally, a 75 IU vial (1 vial containing lyophilized powder + 1 injection containing ready-for-use injection) containing urinary FSH (uFSH) (urofollitropin) (Fostimon^®^, IBSA, Istanbul, Turkey) (n: 6 females; 2 males were added for mating).
- Group 4: experimental group, given 5 IU of Gonal-F intraperitoneally, a 75 IU vial (5.5 µg) containing recombinant FSH (rFSH) (follitropin α),(Gonal-F^®^, Merck Serono, Istanbul, Turkey) (n: 6 females; 2 males were added for mating).

All experimental procedures were conducted in the Laboratory Animal Facility of Manisa Celal Bayar University. Prior to the experiment, the animals underwent a 7-day acclimatization period under controlled environmental conditions. Mice were kept in standard polycarbonate cages (30 × 20 × 13 cm) with stainless steel wire lids and wood shaving bedding. Mice in both the experimental and control groups were housed in standard cages at a constant temperature of 22 °C, relative humidity of 55 ± 5%, and a 14:10 h light–dark cycle (lights on from 07:00 to 21:00). All animals had free access to standard rodent chow and water *ad libitum*.

Two types of FSH preparation, uFSH (5 IU of Fostimon) and rFSH (5 IU of Gonal-F), were used in female mice of Groups 3 and 4 during their estrus cycle. The mice were allowed to mate 48 h after administration of these preparations, and the presence of a vaginal plug was checked daily.

Mice in Group 2 were monitored by vaginal smear to confirm estrus and were allowed to mate spontaneously. The presence of a vaginal plug was also checked daily, and pregnancy was confirmed accordingly. No procedure was carried out on the mice in Group 1, and they were not allowed to mate.

The presence of a vaginal plug was considered day 0.5 of pregnancy. Only females with confirmed vaginal plugs were included in the study. All animals were sacrificed on gestational day 5 at the same predefined peri-implantation time point to ensure synchronization among experimental groups.

### Determination estrous cycle

**Fig. 1:**
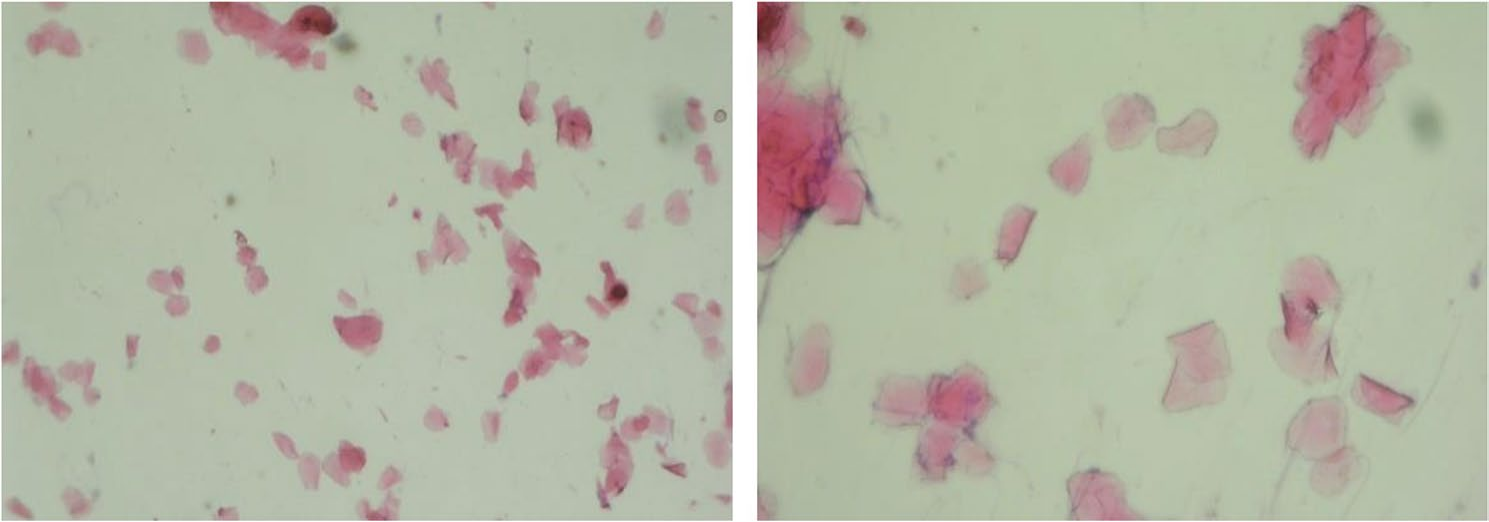
Representative hematoxylin and eosin (H&E) staining of vaginal smear showing the estrous phase of the estrous cycle. The predominance of large, non-nucleated cornified epithelial cells indicates the estrous phase. Original magnifications: ×200 and ×400

The estrus cycle was determined in order to ascertain the execution time of experimental animals. Cotton buds, cover glass, glass objects, hematoxylin–eosin stain and a microscope were prepared for vaginal swab. Cotton buds soaked with 0.9% physiological saline were put into the vaginal opening and rotated 360° to obtain vaginal discharge, which was put on glass objects, dried, and then soaked in methanol 9% for 10 min. It was then stained with haematoxylin for 3 min and with eosin for 2 min. It was then washed in running water and dried, then examined using a microscope with a magnification of 100 times. The results of a vaginal swab for phase determination of mice were based on the presence and quantity of vaginal epithelial cells (Fig. 1) [24].

**Fig. 2:**
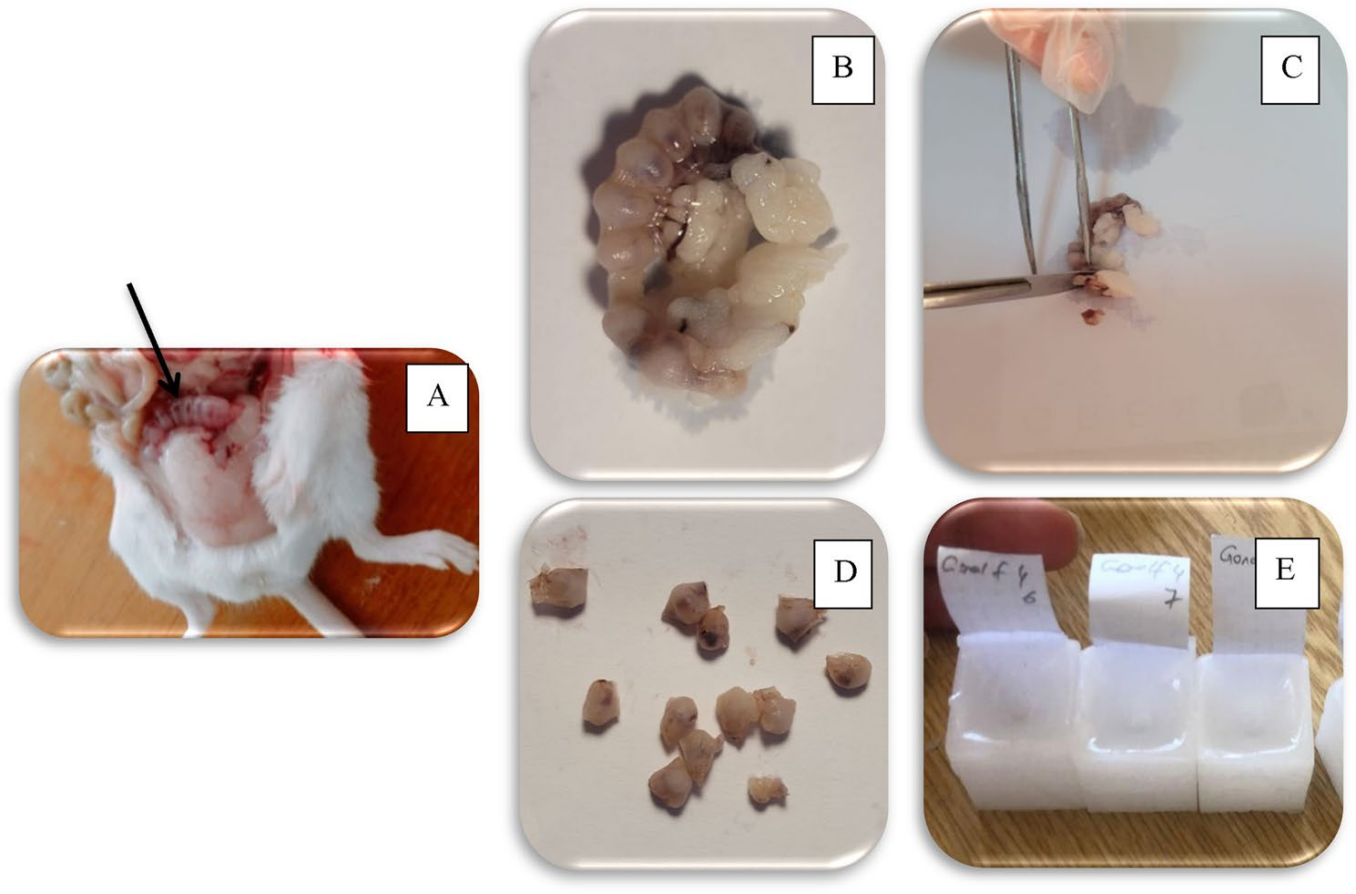
Macroscopic examination of sacrificed mice. Embryos are clearly shown like beads in the horns of the uterus (**A**). Pieces of tissue were taken separately from the horns of the uterus (**B**). These were left for at least 12–48 h in 10% formaldehyde. Before being set in paraffin, the tissues were sectioned with a scalpel (**C**, **D**). After the various procedures, the tissues were immersed in paraffin, placed in block containers and embedded in paraffin (**E**)

Mice were anesthetized intramuscularly with 50 mg/kg ketamine hydrochloride (Ketalar^®^; Pfizer, Istanbul, Turkey) and 5 mg/kg xylazine hydrochloride (Rompun^®^; Bayer Healthcare, Leverkusen, Germany) [25] at the end of the 6th day after coitus. After the study, female mice were sacrificed and their uterus tissues were placed in neutral formalin solution (Fig. 2)

### Haematoxylin–eosin (HE) staining

Paraffin blocks of uterus tissues were produced using a normal light microscope tracking approach. Sections of 5 μm were taken. A total of 100 serial sections from each breast were taken and every 10th, 20th, 30th, 40th, 50th, 60th, 70th, 80th, 90th, and 100th slide was selected.

HE analysis was performed on uterus tissue cross-sections for all animals using image analyzing software (ToupTec Plus Image).

### Immunohistochemical method

Uterus tissue sections were kept at 60 °C overnight for immunohistochemical staining and then cleared with two exchanges of xylene for 30 min. They were then rehydrated for 10 min in distilled water using an alcohol series ranging from 95% to 60%. Sections delimited with an immunohistochemical pen (PatoLab, Japan) were maintained at room temperature for 15 min in a 0.5% trypsin (Thermo Fisher Scientific, Cheshire, UK) solution, and for 5-five minutes to suppress endogenous peroxidase in the tissue. H2O2 was applied at a concentration of 3%. After three five-minute PBS washes, the sections were treated for ten minutes with blocking solution (Thermo Fisher Scientific, Cheshire, UK). After removing the blocking solution from the tissue, primary antibodies against Laminin (1:100, Thermo Scientific, USA), Integrin alpha V beta 3 (1:100, Thermo Scientific, USA) and LIF (1:100, Thermo Scientific, USA) were incubated overnight. The next day, after three PBS washes, the sections were stained for 30 min each with anti-mouse-rabbit biotinylated secondary antibody and streptavidin hydrogen peroxidase (Thermo Fisher Scientific, Cheshire, UK). Between these two stainings and after the last incubation, the sections were washed three times for five minutes with PBS. Finally, 3-Amino-9-Ethylcarbazole (AEC, Spring, CA, USA) was applied for 3–5 min and then washed with PBS. The nuclei were stained with Mayer’s hematoxylin for background staining and then washed with distilled water and mounting media (Spring, CA, USA). Two independent researchers examined immune marker scores and images were acquired using a CX31 light microscope (Olympus, Tokyo, Japan). Ten locations at X400 magnification were randomly chosen for staining in each preparation, and the H score was calculated based on the intensity of the involvement and the percentage of the total amount of involvement. The intensity of involvement was classified semi-quantitatively as 0 (0, no involvement), 1 (+, weak immunoreactivity), 2 (+ +, moderate immunoreactivity), and 3 (+ + +, strong immunoreactivity). The percentage of uptake was calculated using the ratio of immunoreactive cells/structures to total cells/structures; it was scored as 1 (0–10%, focal), 2 (11–50%, regional), or 3 (51–100%, diffuse), and the intensity and amount scores for each area were calculated using the Pi. (i + 1) (Pi = percentage of uptake, I = intensity of uptake) formula. The findings were then added together to get a single value for that slide [25]. Immunohistochemical scoring was performed in predefined endometrial compartments. Laminin and Integrin αVβ3 expression were evaluated in decidualized stromal cells and in the luminal and glandular epithelial cells. LIF immunoreactivity was assessed in glandular epithelial cells, decidual stromal cells, and embryonal structures when present.

For immunohistochemical analyses, appropriate positive and negative controls were included. Peritoneal adhesion tissue was used as a positive control for laminin, integrin, and LIF immunostaining. Negative controls were performed by omitting the primary antibody and replacing it with antibody diluent under otherwise identical conditions. All control and experimental stainings were performed in parallel using identical protocols.

All histological and immunohistochemical evaluations were performed by two independent investigators blinded to the group assignments to ensure unbiased analysis.

### Tail vein blood biochemistry analysis

Whole blood taken via the tail incision method was collected into 5.5 ml tubes with yellow caps and serum separator gel for biomarker analyses. After waiting at least 30 min for the coagulation reaction, the tubes were centrifuged at 1000 g x 15 min at + 4 °C. The serum samples obtained were portioned separately into polypropylene 0.5 ml Eppendorf tubes for each biomarker. Samples were stabilized at −80 °C and stored in a deep freezer until they were used. When the planned number of rats was reached, the serum samples were raised to + 4 °C the night before they were used, and all samples were thawed homogeneously. Analyses of biomarker were performed by the ELISA method with LIF Elisa kit (Finetest, Cambrige, UK). When the number of blood samples reached 24 albino female mice in the whole group, all of them were studied at the same time in order to be statistically significant. Thus, the half-lives of the biomarkers were not affected [26].

### Statistical analysis

When evaluating a reference study in the literature to obtain parameters for sample size estimation, the required sample size for this study was calculated using an ANOVA test with an effect size of 0.6, providing 80% statistical power at a 95% confidence level. The minimum number of participants required per group was determined to be six, resulting in a total of 24 participants [18, 27]. This sample size calculation also accounted for the requirements of other analytical methods planned for the study. The calculation was performed using the G*Power software (version 3.1.9.2, Universität Düsseldorf, Germany). After data collection, statistical analyses were conducted using SPSS software (version 25.0; IBM Corp., Armonk, NY, USA). Data are presented as mean ± standard deviation (SD). The normality of data distribution was assessed using the Shapiro–Wilk test, and homogeneity of variances was evaluated by Levene’s test. Since the data met the assumptions of normality and homogeneity, intergroup comparisons were conducted using one-way analysis of variance (ANOVA), followed by Tukey’s post hoc test to identify pairwise differences. A *p*-value less than 0.05 was considered statistically significant.Statistical significance was set at *p* < 0.05. Post hoc comparisons among groups were conducted using Tukey’s multiple comparison test.

## Results

### Haematoxylin–eosin (HE) staining findings

**Fig. 3:**
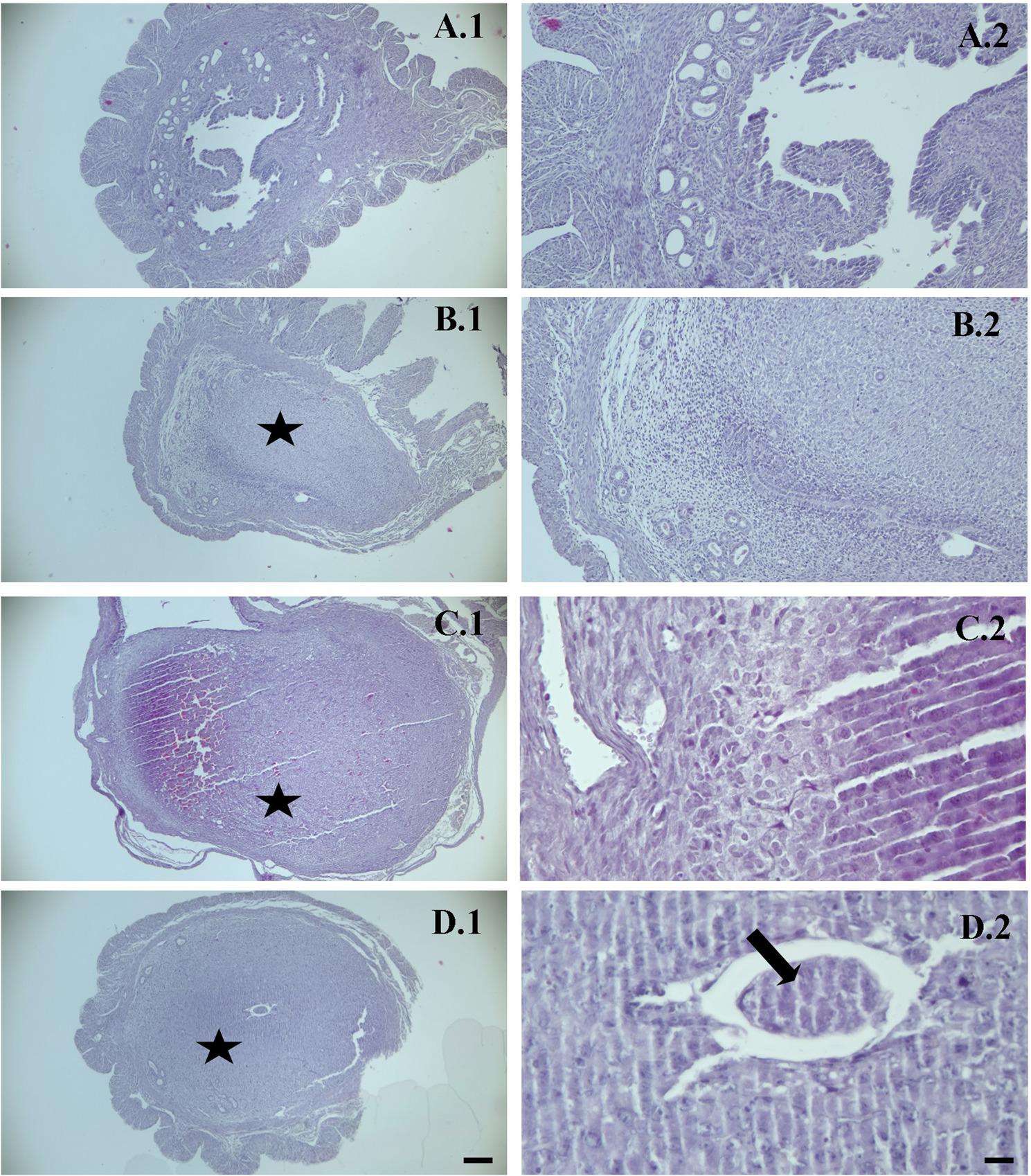
Representative hematoxylin and eosin (H&E) staining of the implantation area in mouse endometrium on day 5 of pregnancy.** A**: Control group, **B**: spontaneous mating group, **C**: uFSH group, **D**: rFSH group. ➨:embryo. ★:Decidualization. The embryo and decidualized stromal regions are indicated. Original magnifications: ×100 (scale bar = 50 μm) and ×400 (scale bar = 10 μm)

In the spontaneous pregnancy, uFSH and rFSH groups in the study, it was found that decidualization had clearly occurred in implantation focus areas, and the embryo had implanted in the uterus. No clear difference was detected in histopathological findings between the groups according to hematoxylin eosin findings (Fig. 3).

### Laminin, integrin alpha V beta 3 and LIF immunostaining findings

**Fig. 4:**
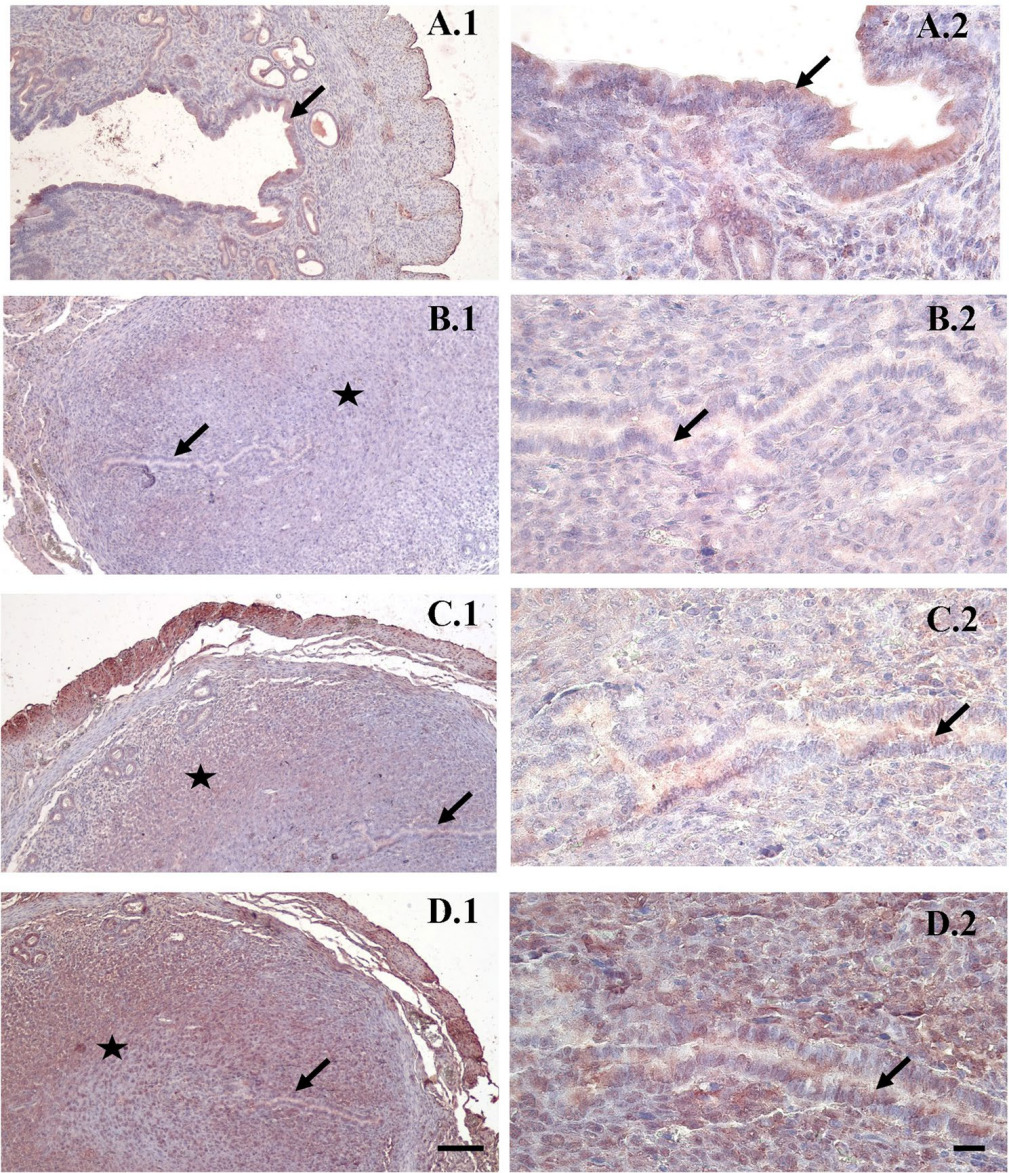
Representative immunohistochemical staining of Laminin in the implantation area of mouse endometrium on day 5 of pregnancy. **A** Control group, **B** spontaneous mating group, **C** uFSH group, **D** rFSH group. ★: Decidualization ➞: Epithelium. Laminin staining is primarily localized in the decidualized stromal region and extracellular matrix, with increased intensity observed in the rFSH-treated group. Decidualization and epithelial regions are indicated. Sections were counterstained with Mayer’s hematoxylin. Original magnifications: ×100 (scale bar = 50 μm) and ×400 (scale bar = 10 μm)

In laminin immunostaining, no decidualization was observed in the control group because there was no pregnancy, and therefore a weak laminin reaction was observed in the epithelium and lamina propria (Fig. 4A1-A2). In the group allowed spontaneous mating, moderate expression was observed in the decidualization area and weak expression in the epithelium (Fig. 4B1-B2). In the uFSH group, moderate expression laminin immunostaining was observed in terms of decidualization and staining intensity in the epithelial region (Fig. 4C1-C2), while in the rFSH group exhibited the most pronounced laminin expression, characterized by moderate to strong staining intensity in the decidualization region (Fig. 4D1-D2, Table 1).

**Table 1:** Mean ± SD and ANOVA F values of immunohistochemical H-scores for Laminin, integrin αVβ3, and LIF across study groups

Anova	Groups	*N*	Mean	Std. Deviation	F	*P*
Laminin	Control	7	108.4	6.2	460.149	0.000*
	Spontaneous	7	223.1	18.1		
	uFSH	7	268.8	3.2		
	rFSH	7	292.7	5.3		
Integrin alpha V beta 3	Control	7	98.4	3.7	2026.654	0.000*
	Spontaneous	7	204	4.3		
	uFSH	7	128.1	3.2		
	rFSH	7	250.2	4.8		
LIF	Control	7	286	4.3	87.115	0.000*
	Spontaneous	7	292	4.3		
	uFSH	7	299.8	4.1		
	rFSH	7	321.2	4.7		

*p<0.00 (Anova), (*) Indicates significant differences among groups

**Fig. 5:**
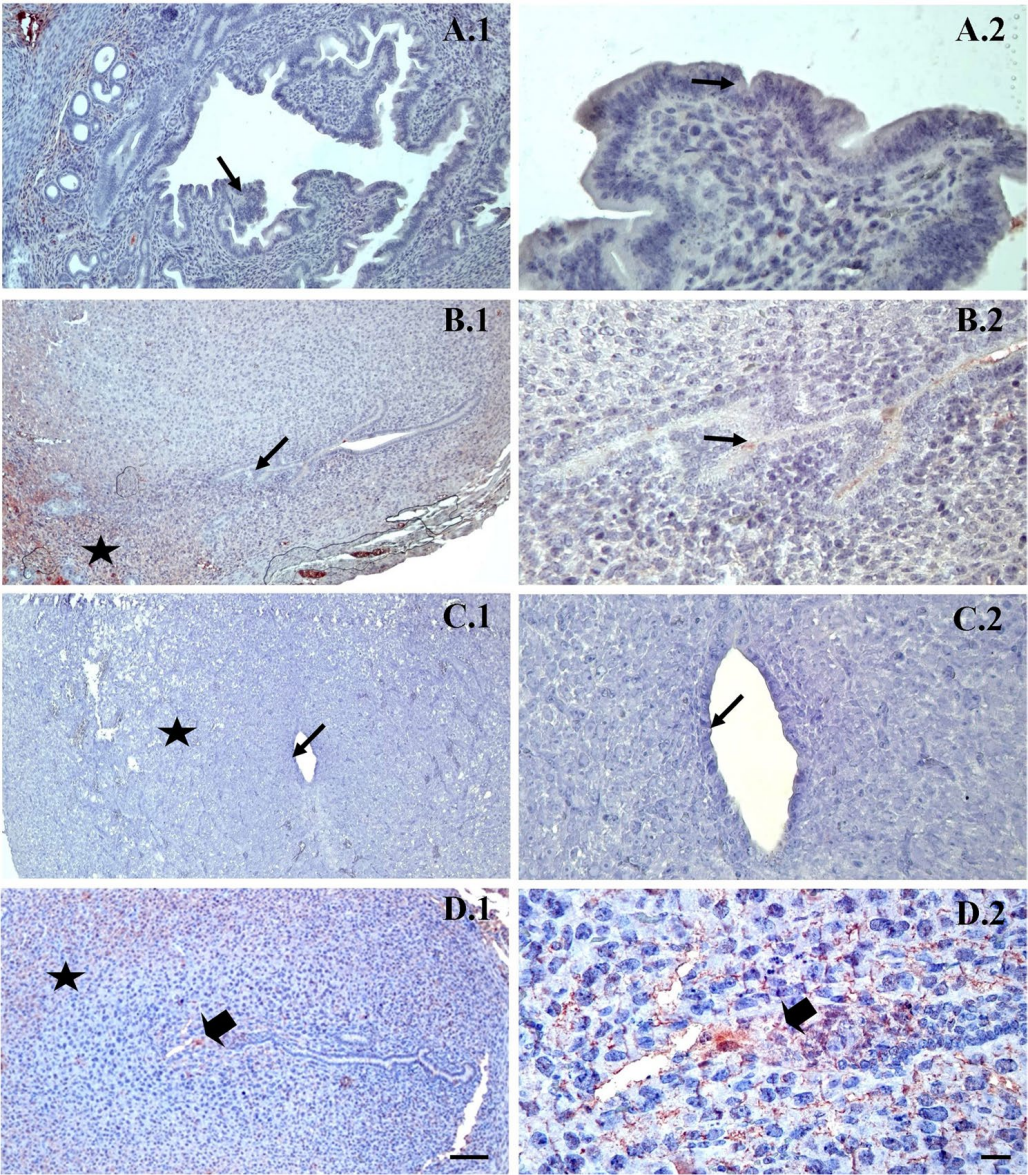
Representative immunohistochemical staining of Integrin αvβ3 in the implantation area of mouse endometrium on day 5 of pregnancy. **A**: Control group, **B**: spontaneous mating group, **C**: uFSH group, **D**: rFSH group. ➨:embryo. ★:Decidualization ➞: Epithelium Integrin staining is predominantly observed along the cell membrane of endometrial epithelial and stromal cells, exhibiting a receptor-type, membranous staining pattern rather than diffuse cytoplasmic labeling. Embryonic and decidualized areas are indicated. Sections were counterstained with Mayer’s hematoxylin. Original magnifications: ×100 (scale bar = 50 μm) and ×400 (scale bar = 10 μm)

In the integrin immunostaining, a weak to moderate reaction was observed in the control group Fig. 5A1-A2), while in the group allowed spontaneous mating, decidualization, and in the lumen epithelium moderate expression, were observed (Fig. 5B1-B2). In the uFSH group, decidualization and a weak reaction with regard to intensity of staining in the epithelium were observed (Fig. 5C1-C2), while in the rFSH group, decidualization, and strong expression with regard to staining in embryonal cells were noted (Fig. 5D1-D2).

**Fig. 6:**
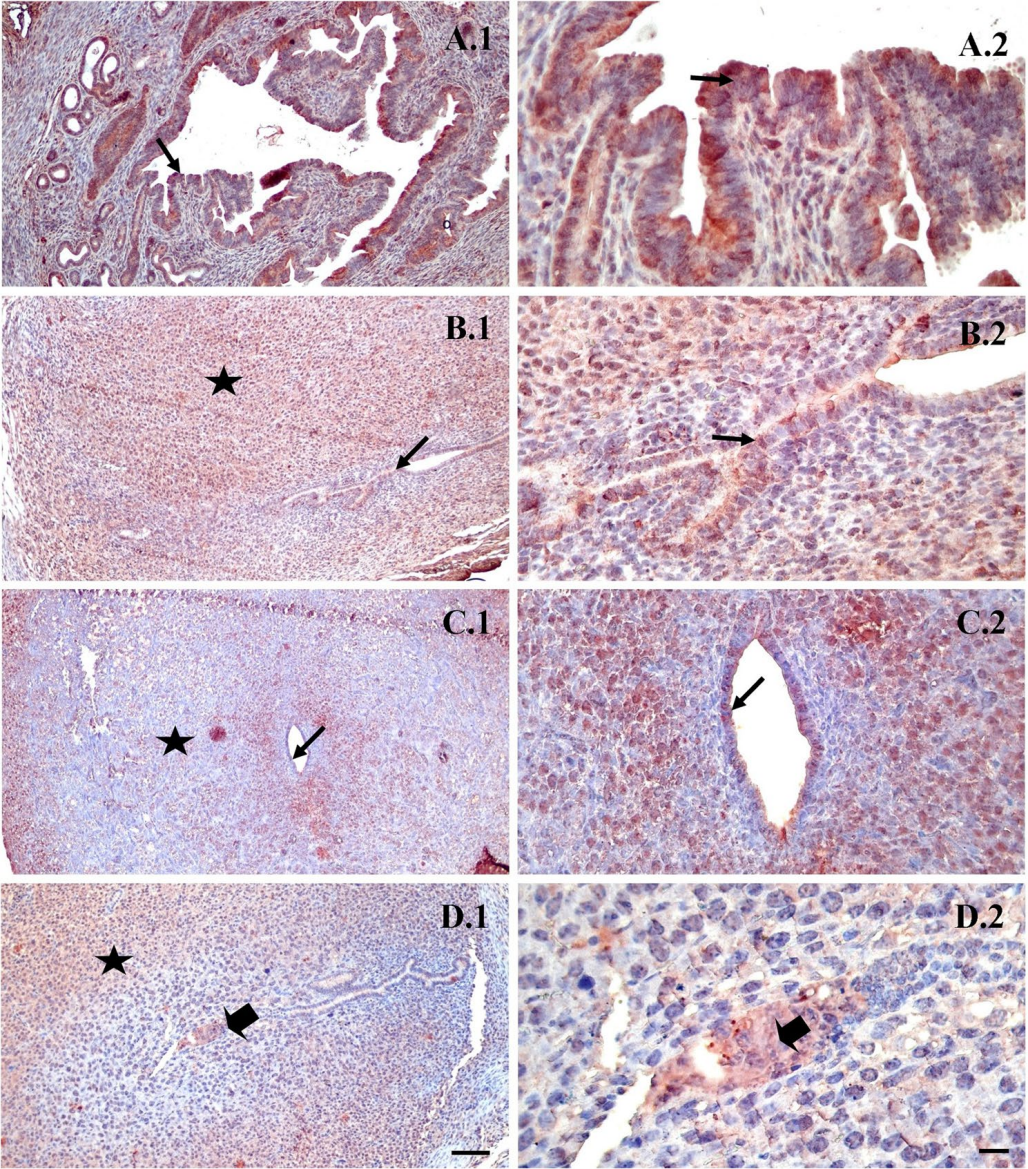
Representative immunohistochemical staining of LIF in the implantation area of mouse endometrium on day 5 of pregnancy. **A**: Control group, **B**: spontaneous mating group, **C**: uFSH group, **D**: rFSH group. ➨:embryo. ★:Decidualization ➝: Epithelium. LIF staining is mainly localized in the cytoplasm of endometrial epithelial cells and glandular structures, with variations in staining intensity among the experimental groups. Embryonic and decidualized regions are indicated. Sections were counterstained with Mayer’s hematoxylin. Original magnifications: ×100 (scale bar = 50 μm) and ×400 (scale bar = 10 μm)

In LIF immunostaining, a strong reaction was determined in the epithelium in the control group (Fig. 6A1-A2). In the spontaneous mating group and in the uFSH group, strong expression was observed in the decidual region and the epithelium (Fig. 6B1-B2, C1-C2), while in the rFSH group, a strong reaction was noted in the decidual region and the embryonal cells (Fig. 6D1-D2).

**Fig. 7:**
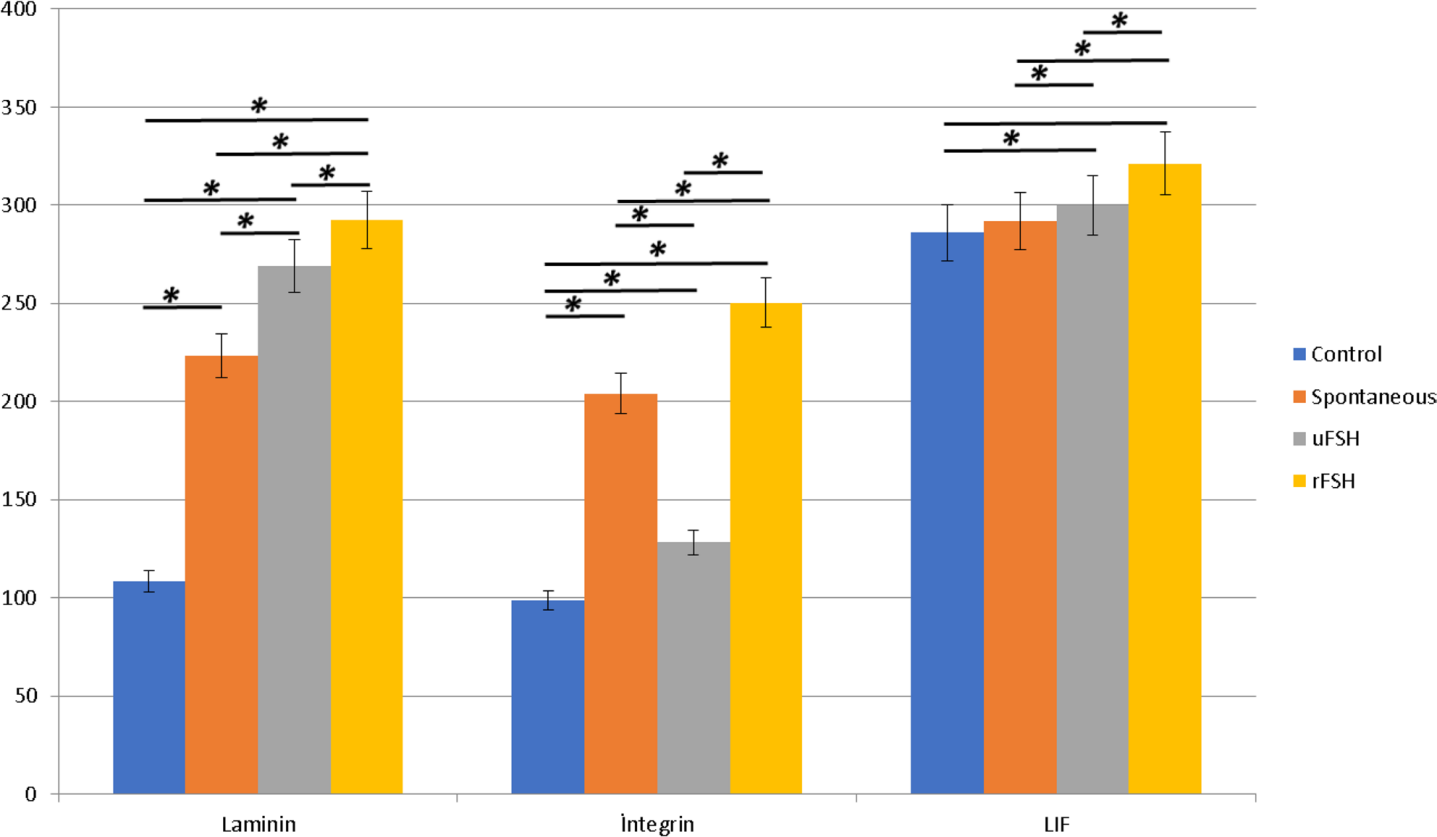
Mean ± SD values of H-scores (morphometric quantification) for Laminin, Integrin αVβ3, and LIF immunostaining among groups. (*) Indicates statistically significant differences between groups

The p-values of Laminin, Integrin αVβ3, and LIF immunohistochemical staining intensities obtained from the post-hoc (Tukey) test were considered statistically significant at *p* < 0.05. According to the multiple comparison results, the staining intensities of Laminin and Integrin αVβ3 showed significant differences among all groups (*p* < 0.001). Specifically, rFSH treatment significantly increased Laminin and Integrin αVβ3 expression compared with both uFSH and spontaneous conception groups (*p* < 0.001), and uFSH administration also resulted in higher expression than spontaneous conception (*p* < 0.001). Regarding LIF expression, no significant difference was observed between the control and spontaneous groups (*p* > 0.05); however, both uFSH and rFSH groups showed significantly higher LIF expression compared with the spontaneous group, with the highest levels observed in the rFSH group (*p* < 0.001) (Tables 1 and 2; Fig. 7).

**Table 2:** Multiple group comparisons between groups in terms of Laminin, Integrin, LIF immunohistochemical density results

Multiple group comparisons between groups *P* Value Posthoc/Tukey	Laminin	Integrin alpha V beta 3	LIF
Control vs. Spontaneous	0.001*	0.000*	0.074
Control vs. uFSH	0.000*	0.000*	0.000*
Control vs. rFSH	0.000*	0.000*	0.000*
Spontaneous vs. uFSH	0.000*	0.000*	0.013*
Spontaneous vs. rFSH	0.000*	0.000*	0.000*
uFSH vs. rFSH	0.000*	0.000*	0.000*

Pairwise Comparisons, Data are *p* values. (*) Indicates significant differences between groups

*p < 0.05 (Anova)

### Tail vein blood LIF biochemical findings

**Fig. 8:**
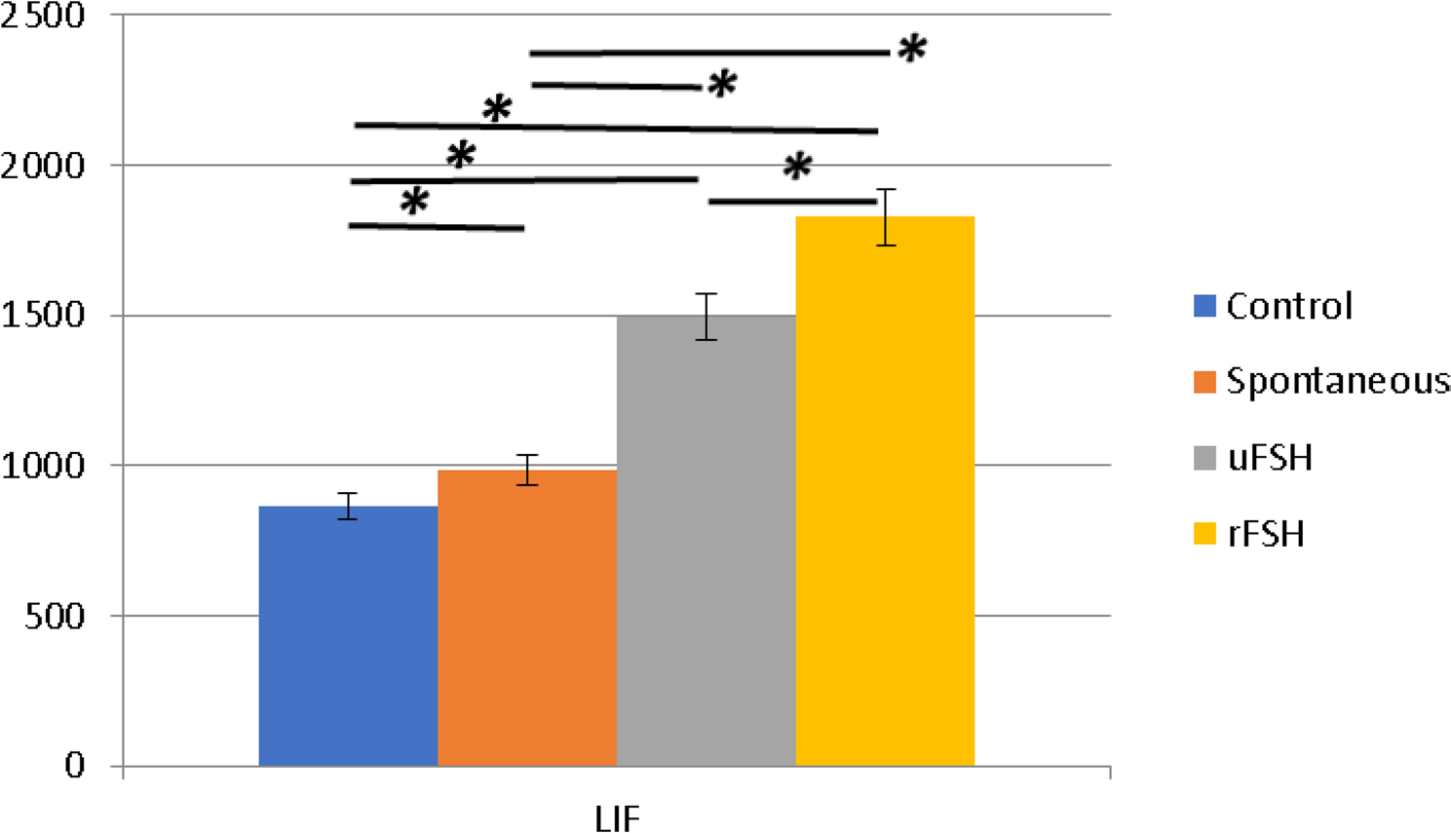
Mean ± SD values of serum LIF concentration (pg/ml) measured by ELISA among groups. (*) Indicates significant differences between groups

Mean ± sd values for the results of LIF biochemical findings between the groups were calculated, and are shown in the table (Table 3). With regard to the results of post hoc (Tukey) comparative analysis, a significant difference was detected in LIF biochemical density among all groups, as *P* values ​​were less than 0.05 (*p* < 0.05) (Fig. 8).

**Table 3:** Mean±SD values for LIF biochemistry results between groups

Anova	Groups	N	Mean	Std. Deviation	F	*P*
LIF (pg/ml)	Control	7	865.2	79.2	4.564	0.011472*
	Spontaneous	7	984.8	104.9		
	uFSH	7	1493.8	468.8		
	rFSH	7	1826.7	998.09		

* p<0.00 (Anova). (*) Indicates significant differences between groups

The mean serum LIF levels of the groups are summarized in Table 3. According to the one-way ANOVA results, a statistically significant difference was observed among the groups (F = 4.564, *p* = 0.011472). The mean LIF concentration was 865.29 ± 79.28 pg/ml in the control group and 984.84 ± 104.98 pg/ml in the spontaneous ovulation group. In the urinary FSH (uFSH)–treated group, the LIF level increased to 1493.86 ± 468.87 pg/ml, while the recombinant FSH (rFSH)–treated group showed the highest mean value (1826.76 ± 998.09 pg/ml). These findings demonstrate that both uFSH and rFSH treatments resulted in markedly elevated serum LIF levels compared to the control and spontaneous groups, suggesting that exogenous FSH administration, particularly recombinant FSH, may enhance endometrial receptivity by upregulating LIF expression.

## Discussion

In the present study, the molecular mechanisms of endometrial receptivity were examined histochemically, immunohistochemically, and biochemically in mice during the estrous phase following ovulation induction with two different FSH preparations (rFSH and uFSH). Uterine tissue samples obtained during the estrous phase were evaluated for Laminin, Integrin αVβ3, and LIF expression using indirect immunohistochemistry. In addition, biochemical analyses of blood samples collected from the tail vein confirmed the effects of FSH administration on ovulation induction, consistent with the experimental groups. Histochemical examinations revealed that decidualization was clearly observed in implantation focus regions, and embryo implantation occurred in all groups, including rFSH, uFSH, and spontaneous mating. Hematoxylin-eosin findings showed no significant histopathological differences among the groups, indicating that the animal model was successfully established and provided a suitable framework for assessing endometrial receptivity markers.

Current clinical guidelines for controlled ovarian stimulation generally consider recombinant FSH and urinary FSH to be clinically comparable in terms of implantation and pregnancy outcomes, and treatment selection is often based on availability, cost, and clinician preference rather than expected differences in endometrial response [28]. However, these recommendations are primarily derived from clinical outcome data and provide limited information regarding potential molecular or histological differences in endometrial receptivity associated with different FSH formulations.The present study was therefore designed to investigate whether different FSH formulations exert distinct effects on key molecular and histological markers of endometrial receptivity beyond clinically observed outcomes. Notably, Laminin expression increased progressively from spontaneous mating to uFSH and rFSH treatment groups, with the highest levels observed following rFSH administration. In contrast, Integrin αVβ3 expression was highest in the rFSH group, whereas uFSH treatment resulted in lower expression compared with spontaneous mating. These findings suggest that although exogenous FSH administration modulates molecular markers of endometrial receptivity, the magnitude and direction of this effect are dependent on the formulation of the gonadotropin, with rFSH exerting a more pronounced impact than uFSH. Similarly, LIF expression increased progressively from spontaneous mating to uFSH and rFSH groups, with the highest levels observed following rFSH administration.

In vitro studies have provided further insight into the mechanisms by which FSH may affect ER. Kim et al. demonstrated that FSH can inhibit the proliferation of mouse uterine endometrial stromal cells in vitro, with rFSH exhibiting stronger inhibitory effects than uFSH. They emphasized the importance of an optimal endocrine environment for in vitro culture of these cells [29]. Similarly, Adonakis and Fleming reported that the negative effects of human menopausal gonadotropin (HMG) and FSH on ER were comparable [30]. Sendag et al. observed in rats that ER markers were lower in both HMG- and rFSH-treated groups compared with natural cycle pregnancies [31]. These findings support the notion that gonadotropins can modulate ER at a molecular level, consistent with the differences observed in laminin, integrin αVβ3, and LIF expression in the present animal study.

Clinical studies examining the effects of rFSH and uFSH on endometrial receptivity are limited, especially when assessed histopathologically. Retrospective and prospective studies suggest that implantation and pregnancy rates are broadly similar between rFSH and uFSH treatments [32, 33]. Pacchiarotti et al. found that combined use of rFSH and uFSH significantly increased oocyte maturation, embryo cleavage, implantation, and pregnancy rates compared with rFSH alone [8]. Despite these findings, clinical studies often lack molecular-level verification of ER markers. It is also known that controlled ovarian hyperstimulation (COH) can negatively affect ER by altering hormonal and molecular endometrial environments [34–36], and that variations in estradiol, progesterone, and testosterone levels may further contribute to these effects [37]. In our study, Integrin αVβ3 expression was lower in the uFSH group compared with the spontaneous mating group, whereas Laminin expression was increased following uFSH administration. This differential pattern may reflect distinct regulatory effects of gonadotropin stimulation on specific molecular components of endometrial receptivity.

Several molecules have been identified as critical for ER and embryo implantation [38]. Rather than relying solely on morphological parameters such as endometrial thickness, increasing evidence indicates that molecular markers provide a more precise assessment of endometrial receptivity during the implantation window [39]. Integrin αVβ3, Laminin, and LIF are among the most studied [40]. Experimental studies demonstrated that integrin αVβ3 mediates embryo attachment and growth in the uterine epithelium [41]. In the study conducted by DuQuesnay et al. in 2009, which aimed to characterize potential endometrial defects in infertile women with isolated polycystic ovary (PCO) morphology, a marked reduction in osteopontin expression—crucial for cell–cell adhesion during the window of implantation—was observed, whereas no alteration was detected in αVβ3 integrin [42]. In the study conducted by Cai et al., the role of αVβ3 integrin in embryo implantation in mice was investigated. The findings indicated that integrin αVβ3 is a critical factor for uterine endometrium–mediated embryo implantation in mice. This integrin, which is distinctly expressed in the mouse blastocyst during the implantation stage, was concluded to facilitate both adhesion and growth processes on uterine epithelial cells, thereby influencing the overall implantation process [43]. In a study conducted by Wang et al., the aim was to evaluate endometrial receptivity during the implantation window in women with unexplained infertility. The authors concluded that among the biomarkers present in uterine fluid, integrin αVβ3 exhibited the highest predictive value for endometrial receptivity [44]. In their 2021 study, Wang et al. reported that, in women with polycystic ovary syndrome (PCOS), endometrial receptivity during the implantation window was more favorable following ovarian stimulation with letrozole (LE) compared with clomiphene citrate (CC). Notably, αVβ3 integrin expression was enhanced in the LE group, which may contribute to the observed increases in clinical pregnancy and ongoing pregnancy rates, highlighting the potential clinical advantages of LE over CC in optimizing endometrial conditions for implantation [45]. Yu et al. investigated the role of N-glycosylation, a critical post-translational modification of glycoproteins, in the establishment of endometrial receptivity. Their study demonstrated that N-glycosylation of integrin αVβ3 and the leukemia inhibitory factor receptor (LIFR) plays a pivotal role in regulating ECM-dependent FAK/Paxillin and LIF-mediated STAT3 signaling pathways, respectively. These findings suggest that by modulating the receptive potential of endometrial cells, N-glycosylation critically influences endometrial receptivity, providing mechanistic insights into implantation processes [46]. Previous studies have consistently reported that integrin αVβ3 is most abundantly expressed in the endometrium of rFSH-induced pregnancies. In the present study, Integrin αVβ3 expression was highest in the rFSH group, whereas uFSH treatment resulted in lower expression compared with spontaneous mating, indicating that gonadotropin stimulation modulates integrin-mediated endometrial receptivity in a formulation-dependent manner. This finding is consistent with experimental and clinical evidence demonstrating the pivotal role of integrin αVβ3 during the implantation window.

Laminin subunits, particularly LAMB3 and Laminin α5, play essential roles in endometrial receptivity and early decidualization [47, 48]. In a study conducted by Li et al., endometrial laminin subunit beta-3 (LAMB3) levels were examined in patients with recurrent implantation failure (RIF) to evaluate their predictive value for pregnancy outcomes. LAMB3 has been proposed as a candidate gene that defines the window of endometrial receptivity in humans. Based on immunohistochemical analysis, the authors concluded that endometrial LAMB3 expression may serve as a promising prognostic marker for predicting pregnancy outcomes in RIF patients undergoing frozen embryo transfer [47]. Emphasizing that the role of laminin alpha-5 in early pregnancy remains incompletely understood, Yang et al. reported that laminin alpha-5 may play a crucial role in the decidualization process in both mice and humans. Yang et al. demonstrated that laminin A5 plays a pivotal role in both mouse and human decidualization. Their findings showed that laminin A5 expression is upregulated during decidualization, is regulated by progesterone via the PKA–CREB–C/EBPβ pathway, and that its knockdown markedly impairs decidualization markers. These results suggest that laminin A5 is a critical regulator of early pregnancy, highlighting its potential importance in the establishment of endometrial receptivity [48]. Previous studies have demonstrated that laminin expression is upregulated during decidualization and contributes to embryo implantation. In our study, Laminin expression was significantly increased in spontaneous mating, uFSH-, and rFSH-treated groups, with the highest expression observed following rFSH administration. Although uFSH treatment enhanced Laminin expression compared with spontaneous mating, this increase remained lower than that observed in the rFSH group. These findings support the notion that laminin-mediated extracellular matrix remodeling during implantation is sensitive to the source and formulation of gonadotropin stimulation.

LIF, secreted by uterine glands, is crucial for stromal cell decidualization and embryo implantation [49–51]. According to Xiao et al., Yikang decoction therapy may regulate integrin αVβ3 expression and upregulate LIF expression, thereby enhancing cellular adhesion and ultimately improving embryo implantation in mice [52]. In another study, Hosseini et al. evaluated the effect of LIF—recognized as one of the most critical trophic factors in early embryonic development—on human endometrial epithelial cells (hEECs). Although hEECs secreted LIF during co-culture with embryos, the concentration was insufficient; thus, the authors suggested that supplementation with exogenous LIF may enhance embryonic developmental potential [53]. Similarly, Zarei et al. (2020) reported that type 2 diabetes may contribute to implantation failure and emphasized the uncertainty regarding the effects of antidiabetic agents on implantation. Their study assessed alterations in VEGFA and LIF expression during implantation in diabetic rats treated with metformin or pioglitazone. The results demonstrated decreased VEGFA and LIF expression in diabetic rats, potentially contributing to diabetes-related infertility, while pioglitazone was more effective than metformin in restoring VEGFA and LIF expression to near-reference levels [54]. Dhakal et al. investigated the effects of uterine glands on embryo survival and stromal cell decidualization in mice. Their findings strongly support the hypothesis that uterine glands secrete not only LIF but also additional factors that contribute to embryo survival and the decidualization process essential for successful pregnancy [55]. In line with this, Hosseini et al. reported that blocking LIF exerts a detrimental effect on embryo implantation in mice and demonstrated that LIF regulates endometrial integrin αVβ3 expression. They further showed that LIF promotes blastocyst growth and cell proliferation, upregulates integrin alpha V (Itgav) and integrin beta 3 (Itgb3) gene expression, and plays a crucial role in the success of implantation [56]. Previous research has consistently highlighted the pivotal role of leukemia inhibitory factor (LIF) in establishing uterine receptivity and enabling successful embryo implantation. In murine models, Terakawa et al. demonstrated that uterine epithelium-specific deletion of the LIF receptor (LIFR) completely abolished implantation, underscoring the indispensable nature of LIF/LIFR-ERBB2 signaling in early pregnancy [57]. Complementing this, Li et al. reported that LIF promotes proliferation of endometrial epithelial cells and regulates key receptivity-associated genes such as HOXA10, indicating that basal expression of LIF contributes to a primed endometrial state even prior to implantation [58]. Clinical relevance has been further emphasized by Fukui et al., who identified LIF expression in the cervix as a non-invasive biomarker that mirrors uterine receptivity and correlates with implantation capacity [59]. At the molecular level, Salmasi et al. showed that microRNA-181 directly targets LIF transcripts, thereby impairing its expression and reducing implantation potential, highlighting post-transcriptional regulation as an additional layer of control [60]. Finally, Paulson et al. provided a broader perspective, reviewing evidence from rodent models and noting that while LIF plays a central role in these species, its translational application across other mammals remains less well established [61]. In line with previous evidence, our findings reinforce the notion that LIF is a pivotal regulator of endometrial receptivity and embryo implantation. Notably, LIF expression was observed in the endometrium even in the absence of pregnancy, suggesting basal secretion by uterine glands. While no statistically significant difference was observed between the control and spontaneous pregnancy groups (*p* > 0.05), both uFSH and rFSH stimulation resulted in significantly increased LIF expression, with the highest levels observed in the rFSH group. These results indicate that gonadotropin stimulation may enhance uterine gland function and embryo–endometrium signaling through upregulation of LIF. The present study confirms that rFSH induces the highest expression of endometrial receptivity markers, supporting a positive effect on implantation potential. uFSH treatment resulted in moderately increased Laminin expression compared with spontaneous conception; however, these levels remained significantly lower than those induced by rFSH. In contrast, Integrin αVβ3 expression was lower in the uFSH group than in the spontaneous mating group, while the highest expression was observed following rFSH administration. These findings highlight potential discrepancies between molecular, histopathological, and clinical observations and underscore the importance of integrating molecular data into assisted reproductive technology outcomes [62].

This study has several limitations. The use of a *Mus musculus* model limits the direct extrapolation of the findings to human implantation physiology. Although animals were randomized and evaluations were performed in a blinded manner, subtle environmental and biological variables cannot be completely excluded. Marker expression was assessed at a single peri-implantation time point, which may not fully reflect the dynamic temporal regulation of endometrial receptivity markers. In addition, the sample size was calculated based on expected differences in LIF expression; therefore, smaller effect sizes for laminin and integrin αVβ3 may have remained undetected. Furthermore, due to budgetary constraints, the scope of the present study was limited to tissue-based immunohistochemical analyses. Among the evaluated markers, priority was given to LIF, as it is a well-established implantation marker expressed by both embryonic and maternal tissues during the peri-implantation period. Consequently, systemic evaluation of other markers, such as circulating laminin and integrin αVβ3 levels, was beyond the scope of this study and is planned to be addressed in future independent research. Despite these limitations, the consistency of the histochemical, immunohistochemical, and biochemical findings supports the conclusion that rFSH and uFSH exert differential effects on molecular markers of endometrial receptivity.

## Conclusion

Successful fertilization alone is insufficient for the establishment of a healthy pregnancy; implantation depends on the precisely synchronized interaction between a receptive endometrium and a developmentally competent embryo. Although the molecular mechanisms underlying endometrial receptivity (ER) remain incompletely understood, the present findings provide comparative evidence that ovulation induction with recombinant FSH results in the highest expression of key receptivity markers, including Laminin, Integrin αVβ3, and LIF. Both uFSH and rFSH treatments were associated with increased expression of Laminin and LIF compared with spontaneous conception, whereas Integrin αVβ3 expression was reduced following uFSH treatment but markedly increased after rFSH administration. These findings indicate that exogenous gonadotropin stimulation modulates molecular features of endometrial receptivity in a formulation-dependent manner, with rFSH consistently exerting a stronger effect than uFSH. Collectively, these results suggest that recombinant FSH may promote a more favorable endometrial molecular environment for implantation than urinary FSH. Taken together, the findings underscore the importance of prospective clinical studies directly comparing rFSH, uFSH, and natural cycles, integrating molecular, histopathological, and clinical outcomes to better elucidate implantation mechanisms and optimize assisted reproductive technology strategies.

## Supplementary Information

Supplementary Material 1.

## Data Availability

The datasets used and/or analysed in the current study are available from the corresponding author upon reasonable request.

## Abbreviations

GnRH: Gonadotropin-releasing hormone
HMG: Human menopausal gonadotropin
rFSH: Recombinant FSH
uFSH: Urinary FSH
ART: Artificial reproductive technologies
integrin αVβ3: Integrin alpha V beta
LIF: Leukemia Inhibitory Factor
WOI: Window of implantation
